# Doxorubicin-induced acute cardiotoxicity is associated with increased oxidative stress, autophagy, and inflammation in a murine model

**DOI:** 10.1007/s00210-023-02382-z

**Published:** 2023-01-16

**Authors:** Patricia Lorena Dulf, Mihaela Mocan, Camelia Alexandra Coadă, Daniel Vasile Dulf, Remus Moldovan, Ioana Baldea, Anca-Daniela Farcas, Dan Blendea, Adriana Gabriela Filip

**Affiliations:** 1 Iuliu Hatieganu University of Medicine and Pharmacy, 400012 Cluj-Napoca-Napoca, Romania; 2 Emergency Clinical County Hospital, 40006 Cluj-Napoca-Napoca, Romania; 3 Department of Internal Medicine, Iuliu Hatieganu University of Medicine and Pharmacy, 400012 Cluj-Napoca-Napoca, Romania; 4 Department of Medical and Surgical Sciences (DIMEC), University of Bologna, 40138 Bologna, Italy; 5 Department of Molecular Sciences, Iuliu Hatieganu University of Medicine and Pharmacy, 400394 Cluj-Napoca-Napoca, Romania; 6 Medisprof Cancer Center, 400641 Cluj-Napoca-Napoca, Romania; 7 Department of Functional Biosciences, Iuliu Hatieganu University of Medicine and Pharmacy, 400012 Cluj-Napoca-Napoca, Romania; 8 Department of Cardiology, Heart Institute, 40001 Cluj-Napoca-Napoca, Romania

**Keywords:** Cardiotoxicity, Chemotherapy, Doxorubicin side-effects, Oxidative stress, Autophagy, Ultrasonography

## Abstract

Drug-induced cardiotoxicity is a life-threatening side effect of doxorubicin (DOX) treatment that impacts patient prognosis and survival. In the majority of cases, the acute clinical form often remains asymptomatic, with few patients presenting rather nonspecific electrocardiographic abnormalities. While chronic toxicity has been more widely studied, the alterations appearing in acute cardiotoxicity are much less investigated. Thus, our in vivo study aimed to evaluate the process of DOX-induced acute myocardial toxicity by investigating oxidative stress and autophagy markers as mechanisms of myocardial toxicity in correlation with echocardiography and electrocardiography findings. Our results show that both autophagy and oxidative homeostasis were disrupted as soon as 7 days after DOX treatment, alterations that occurred even before the significant increase of NT-proBNP, a clinical marker for cardiac suffering. Moreover, we found a large number of alterations in the electrocardiography and echocardiography of treated rats. These findings suggest that DOX-induced myocardial toxicity started early after treatment initiation, possibly marking the initial phase of the unfolding process of cardiac damage. Further studies are required to completely decipher the mechanisms of DOX-induced cardiotoxicity.

**Graphical Abstract:**

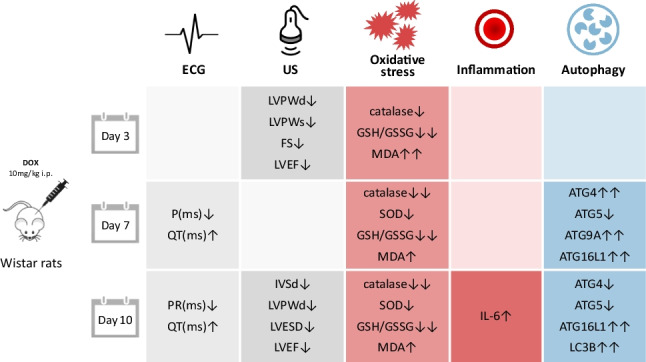

## Introduction

In the past years, cancer relative survival rates have increased significantly as a consequence of the breakthroughs in cancer therapy (Siegel et al. 2020). The Early Breast Cancer “Trialists” Collaborative Group (EBCTCG) reported that the inclusion of anthracyclines in the management of breast cancer improved absolute survival with approximately 3% at 5 years and 4% at 10 years. Since then, anthracyclines remained the cornerstone of treatment for breast cancer patients together with the recently discovered checkpoint blockade immunotherapy and targeted therapies (Bines et al. 2014; Early Breast Cancer Trialists’ Collaborative Group (EBCTCG) et al., 2012). However, anthracycline-contained regimens such as doxorubicin (DOX) are not devoid of severe side effects such as anthracycline-induced cardiotoxicity (AIC) which can even result in the patients’ death (Bansal et al. 2019). Moreover, cardiotoxicity is usually progressive and irreversible; thus, the identification of AIC signs as early as possible is of great interest in the field of cardio-oncology, so that cardioprotective strategies can be employed early on to reduce cardiovascular mortality and prevent the interruption of antineoplastic therapy (Curigliano et al. 2020; Hinrichs et al. 2020).

Cardiotoxicity is divided into acute, subacute, chronic, and late, based on the time elapsed between the drug administration and the first sign of toxicity. While chronic toxicity has been more widely studied, the manifestations that appear in acute cardiotoxicity are much less investigated, and the progression to a secondary dilated cardiomyopathy is not fully understood (Cardinale et al. 2020; Veronese et al. 2018). Even though myocardial injury may begin straight after drug administration, the acute clinical form often remains asymptomatic, with only around 11% of patients presenting some electrocardiographic abnormalities such as QT interval prolongation and nonspecific ventricular repolarization changes (Monsuez et al. 2010; Pai and Nahata 2000). ECG and echocardiography, along with serum biomarkers such as NT-proBNP, are practical non-invasive tools for measuring cardiac function and morphology in a clinical setting and are recommended by international guidelines (Plana et al. 2014; Zidan et al. 2015). Numerous pathways have been investigated in the context of AIC, but the complete mechanisms are yet to be fully elucidated. Some studies show the harmful effects of DOX on cardiomyocytes are caused by consequent mitochondrial dysfunction with the generation of reactive oxygen species (ROS) (Nordgren and Wallace 2020; Sardão et al. 2009). Because cardiomyocytes have a relatively lower level of antioxidant enzymes such as superoxide dismutase (SOD) and catalase (C. Pereira et al. 2011), they are more susceptible to ROS-induced lipid peroxidation and subsequent oxidative damage (“Doxorubicin-induced Apoptosis in Endothelial Cells and Cardiomyocytes Is Ameliorated by Nitrone Spin Traps and Ebselen - Journal of Biological Chemistry,” n.d.; Tsang et al. 2003). Investigations of autophagy as the underlying mechanism of AIC rendered conflicting results (Dirks-Naylor 2013; Koleini and Kardami 2017; Xu et al. 2012; Zhang et al. 2009). Thus, several studies suggest that DOX contributes to the pathogenesis of AIC through the upregulation of cardiac autophagy (Katamura 2014; Xu et al. 2012), while others support the notion that suppression of autophagy is the main culprit (Christidi and Brunham 2021).

Based on these data, the aim of our study was to evaluate oxidative stress and autophagy as mechanisms of in vivo acute cardiac toxicity induced by DOX treatment, which was diagnosed by electrocardiographic and echocardiographic changes.

## Materials and methods

### Animal models

A total of 30 adult male Wistar albino rats weighing 150–250 g were provided by the Animal Department of the “Iuliu Hațieganu” University of Medicine and Pharmacy, Cluj-Napoca, Romania. They were housed in the designated animal facility, in an air-conditioned room, with temperatures maintained at 23±2 °C and a 12-h dark/light cycle and received food and water ad libitum. The number of animals was chosen to ensure the optimal balance between having a sufficient number of samples to achieve statistical significance and keeping it ethically minimal. Sample size calculation was performed based on previous pilot studies, using a beta of 0.8 and an alpha of 0.05.

### Experimental design protocol

**Fig. 1:**
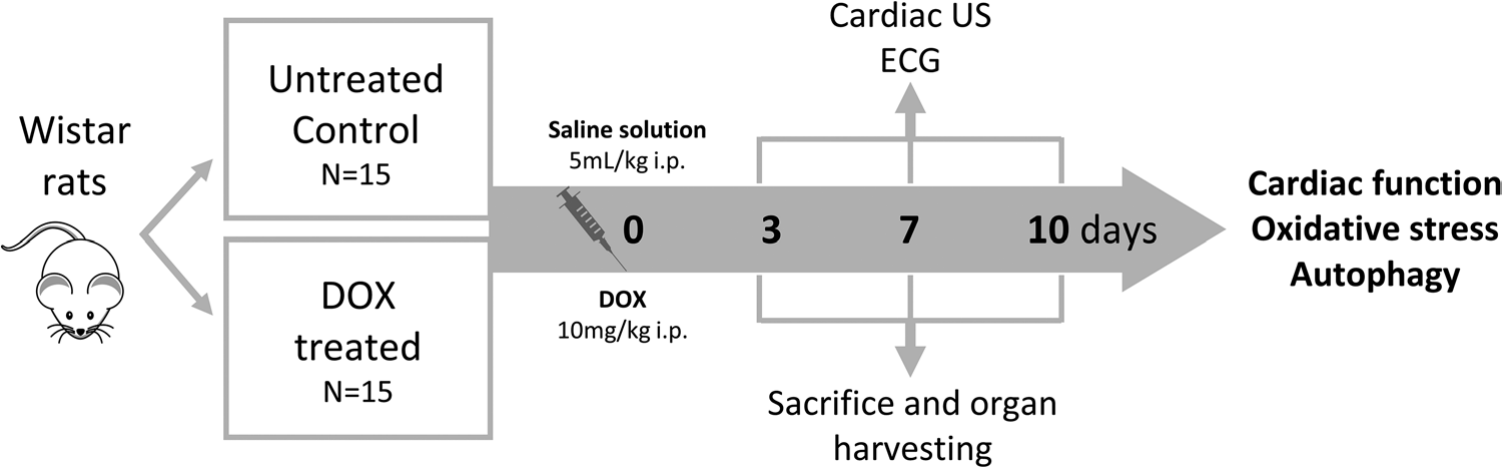
Study workflow. Thirty rats were randomly divided into DOX-treated and untreated groups and received a single intraperitoneal injection of doxorubicin or saline solution respectively. At 3, 7, and 10 days, rats were subjected to cardiac US and ECG and sacrificed for sample harvesting

Thirty rats were randomly divided into two groups: DOX group—animals received a single intraperitoneal injection of 10 mg/kg Doxorubicin (Sigma–Aldrich Chemicals GmbH, Germany) and a control group which received a single intraperitoneal injection of 5 mg/kg saline solution (Hayward and Hydock 2007). A schematic representation of the protocols and methods can be found in Fig. 1. The time points were selected based on data from pharmacokinetic studies showing that the average half-life of doxorubicin lies between 12 and 48 h (Sun et al. 2017); thus, electrocardiography (ECG) followed by echocardiography were performed at 3, 7, and 10 days. At these time points, rats were euthanized under anesthesia with ketamine and xylazine (90 mg/kg b.w.; 10 mg/kg b.w.). The blood, heart, liver, spleen, and kidneys were harvested from each rat for consecutive analyses.

### Electrocardiography and echocardiography

ECG was recorded using a Biopac MP36 system (Goleta, CA, USA), designed for lab animal ECG interpretation, as previously described (Balea et al. 2018; Boarescu et al. 2019a, b; Boarescu et al. 2019a, b). The rats were anesthetized with an intraperitoneal injection of ketamine (26 mg/kg of body weight) and xylazine (2.6 mg/kg of body weight) (Filip et al. 2021).

Fifteen minutes after anesthesia, electrodes were attached to the paw pads of each rat, and ECG was recorded in the lead II. RR and QT intervals, PR segment, QRS complex duration (ms), and ST-segment changes (mV) were calculated from ECG recordings using the Biopac Student Lab 3.7.7 software (Goleta, CA, USA); the paper speed was 50 mm/s. Heart rates (HR, beats/min) were calculated from the RR intervals according to the following formula: HR = 60,000/RR. Corrected QT intervals (QTc) (ms) were calculated according to the Bazett formula (Dahlberg et al. 2020).

Echocardiography is a practical non-invasive tool for measuring cardiac function and morphology both in a clinical setting but also in animals (Migrino et al. 2008) and is the most used method to detect DOX-induced cardiotoxicity by assessing cardiac function (Anqi et al. 2019). Therefore, for rat cardiac ultrasound (US), the anterior chest hair was shaved, and rats were positioned in left lateral decubitus allowing for ECG monitoring throughout the experiment. The temperature was kept at 37 °C with a heated plate. Prewarmed ultrasonography gel was applied to the thorax prior to the application of the imaging probe. The examination was carried out under spontaneous ventilation by two different operators with an Ultrasonix ultrasound machine, using a 15–20 Hz phased array probe (12S-RS). The transmission frequency was 15 MHz, the depth was 1.5 cm, and the frame rate was 25 frames per second. Measurements were performed in accordance with the leading-edge method of the American Society of Echocardiography (Lang et al. 2005).

All measurements were made from more than three beats and averaged.

Two-dimensional short-axis imaging using M-mode echocardiography of the left ventricle (LV) at the level of the papillary muscle was used to determine the internal diameters of the left ventricles during systole and diastole, to calculate the fractional shortening (%FS), which was used as an index of systolic function, and to calculate the left ventricular ejection fraction (LVEF) by using the Teichholz method (Wilson et al. 1998, Chengode 2016). In addition, LV posterior and septal wall thickness were measured and used to calculate the LV mass. A single investigator performed all measurements. A second investigator, who was also experienced in the echocardiographic analysis of rodent hearts, reviewed all measurements. The LV end-diastolic dimension (LVDd) was measured at the maximal diastolic dimension, and the LV end-systolic dimension (LVDs) was measured at the maximal anterior motion of the posterior wall. Measurements represented the mean of five consecutive cardiac cycles using the same transducer position and angle in the same stop image frame.

### Oxidative stress assessment

For quantification of redox levels in the heart, liver, kidney, and spleen, the malondialdehyde (MDA) as a marker of lipid peroxidation was evaluated at 3, 7, and 10 days using the Conti method (Conti et al. 1991). In addition, reduced glutathione (GSH), oxidized glutathione (GSSG), ratio GSH/GSSG, superoxide dismutase, and catalase activities were assessed in the heart tissue. Superoxide dismutase activity was determined using the cytochrome *c* reduction test as described by Beauchamp and Fridovich 1971 (Beauchamp and Fridovich 1971). Catalase and GSH/GSSG ratio were measured as previously described (Pippenger et al. 1998). The analyzed tissues were homogenized with polytron homogenizer using Tris buffer solution as previously described by Mitrea et al. 2020 (Mitrea et al. 2020), and the protein levels were evaluated using the Bradford method (Sigma–Aldrich Chemicals GmbH, Germany).

### Autophagy and inflammation evaluation in the heart tissue

Western blot analysis was used for the evaluation of autophagy proteins. Total protein lysates (40 µg/lane) were separated by electrophoresis on SDS PAGE gels and then transferred to PVDF membranes, using the Bio-Rad Miniprotean system (Bio-Rad). Blots were blocked with StartingBlock Buffer (Thermo Fischer Scientific) and then incubated with primary antibodies against Beclin 1, ATG4b, ATG5, ATG9a, ATG16L1, LC3B (Abcam ab228525), IL-6 (Abcam), and GAPDH (Santa Cruz; sc-166545). Detection was done using Supersignal West Femto-Chemiluminiscent substrate (Thermo Fisher Scientific, Rockford, IL, USA). Image acquisition was done using ChemiDoc System (Bio-Rad) while quantification was done using Image Lab analysis software (Bio-Rad, Hercules, CA, USA). GAPDH was used as a protein loading control. All investigated proteins were normalized on GAPDH after which DOX-treated samples were represented as a fold change of their respective untreated controls.

### Statistical analysis

Statistical analysis was performed using GraphPad Prism 8 (GraphPad Software Inc., San Diego, CA, USA). Continuous variables with a normal distribution were expressed as mean ± standard deviation (SD). Variables were analyzed using the Student *t*-test and ANOVA test as appropriate. A value of *p*<0.05 was considered significant.

## Results

There were no recorded deaths in any of the experimental animal groups during the study period.

### Effects of DOX on cardiac electrophysiology

First, in order to get a general overview of DOX-induced cardiac damage, we measured the ECG tracings at 3, 7, and 10 days. Rhythm disorders were assessed by measuring the RR interval, heart rate (HR), PR, QRS, and QT intervals (Table 1). The RR, PR interval, and QRS duration were not significantly altered between groups (*p*>0.05) (Table 1). At 7 days, there was a statistically significant difference between the amplitude of the P waves between the two groups (35.6±4 vs 31.2±3.9; *p*=0.037) as well as between the QT and QTc intervals duration (Table 1). Moreover, ST segment depression was also seen in the DOX group at this time point (0.027±0.007 mm). In addition, at 10 days, DOX-treated rats showed a decreased PR interval (55.2±1.9 vs 42±13.6; *p*=0.04).

**Table 1:** ECG parameters of DOX-treated and -untreated rats. *HR*, heart rate (beats/min); *R-R*, RR interval; *P*, P wave; *P-R*, PR interval; *QRS*, QRS interval; *QT*, QT interval; *QTc*, corrected QT interval

Variable	Days	Mean ± SD		*p*-value
		NT	DOX	
HR (b/min)	3	251.2 ± 35.8	285.2 ± 43.3	0.25
	7	245.6 ± 35.4	277.8 ± 61.6	0.32
	10	256.8 ± 21.8	266.8 ± 60.7	0.75
P (ms)	3	32.2 ± 1.4	29.4 ± 2.6	0.17
	7	35.6 ± 4	31.2 ± 3.9	**0.037**
	10	34.2 ± 1.6	32.8 ± 3.9	0.48
P-R (ms)	3	56.2 ± 3.8	53 ± 8.3	0.61
	7	58.6 ± 6.4	52 ± 16.4	0.3
	10	55.2 ± 1.9	42 ± 13.6	**0.04**
R-R (ms)	3	290.2 ± 72	257.4 ± 75.2	0.33
	7	248.6 ± 36.8	224 ± 48.2	0.38
	10	241.6 ± 22.3	222 ± 38.3	0.49
QRS (ms)	3	32.4 ± 2.7	32.8 ± 2.5	0.98
	7	31.4 ± 1.1	31 ± 0.7	0.98
	10	31 ± 1.2	31.6 ± 2.5	0.85
QT (ms)	3	75 ± 7.1	76.2 ± 7.9	0.8
	7	69.2 ± 3.9	81 ± 13.3	**0.02**
	10	64.6 ± 2.5	76.2 ± 4.8	**0.03**
QTc	3	160 ± 16.3	152.8 ± 22.2	0.51
	7	142 ± 9.5	177 ± 20.4	**0.008**
	10	133.7 ± 8.6	160 ± 9.9	**0.03**

*p*-values wrtten in bold were significant

### Effects of DOX on cardiac function and cardiac remodeling

**Fig. 2:**
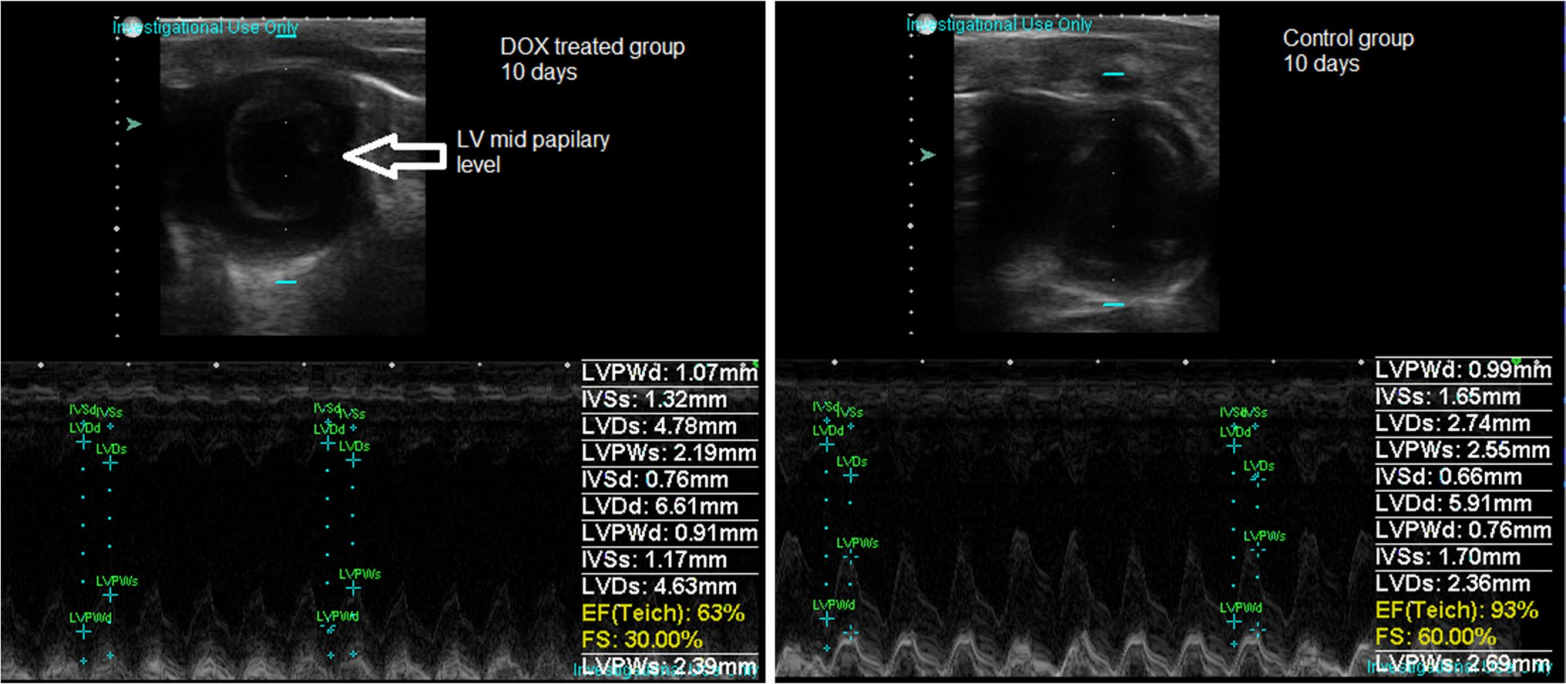
Representative image of rat heart ultrasound (US). Cardiac function evaluated by echocardiography. M-Mode imaging with the measurements of diastolic left ventricular internal dimensions (LVIDd), systolic left ventricular internal dimensions (LVIDs), interventricular septal end diastole (IVSd), and systole (IVSs) and left ventricular posterior wall end diastole and (LVPWd) and systole (LVPWs), left ventricular ejection fraction (EF) calculated after the Teicholz method, shortening fraction (FS) in control (**A**) vs DOX-treated (**B**) rats at 10 days

The acute effects of doxorubicin administration on cardiac function and geometry were evaluated by means of M-mode echocardiography at 3, 7, and 10 days. As soon as 3 days after DOX administration, the rats began to show alterations of normal cardiac anatomy and activity as shown on US (Table 2). Namely, left ventricle (LV) posterior wall thickness was lower both in diastole as well as in systole (2.1±0.5 vs 1.3±0.2; *p*<0.002, 3.4±0.5 vs 2.3±0.3; *p*<0.001 respectively). Moreover, at 10 days, DOX treatment led to the dilation of the left ventricle as evidenced by an increased LV end-systolic dimensions (2.4±0.2 vs 3.3±0.9; *p*=0.04). LV end-diastolic dimension at this time point was close to attaining statistical significance (*p*=0.06). Ejection fraction was significantly lower at 3 and 10 days (89.6±1.1 vs 80.4±5.1; *p*=0.03, 89.8±2.1 vs 81.4±10.8; *p*=0.05 respectively) (Table 2, Fig. 2).

**Table 2:** US parameters of DOX-treated and untreated rats. *LV*, left ventricle; *IVSd*, interventricular septal thickness in diastole; *IVSs*, interventricular septal thickness in systole; *LVPWs*, LV posterior wall thickness in diastole; *LVPWd*, LV posterior wall thickness in systole; *LVEDD*, LV end-diastolic dimension; *LVESD*, LV end-systolic dimension; *LVEF*, LV ejection fraction; *FS*, fractional shortening. p-values wrtten in bold were significant.

Variable (mm)	Days	Mean ± SD		*p*-value
		NT	DOX	
IVSd	3	1.2 ± 0.2	1.3 ± 0.2	0.99
	7	1.3 ± 0.1	1 ± 0.1	0.09
	10	1.5 ± 0.2	0.8 ± 0.1	** < 0.001**
IVSs	3	2.3 ± 0.3	2.3 ± 0.3	0.99
	7	2.3 ± 0.1	1.7 ± 0.8	0.12
	10	2.7 ± 0.2	1.9 ± 0.3	**0.04**
LVPWd	3	2.1 ± 0.5	1.3 ± 0.2	** < 0.002**
	7	1.3 ± 0.1	1.2 ± 0.2	0.95
	10	1.5 ± 0.1	1.1 ± 0.3	**0.05**
LVPWs	3	3.4 ± 0.5	2.3 ± 0.3	** < 0.001**
	7	2.4 ± 0.2	2.4 ± 0.4	0.99
	10	2.4 ± 0.1	2.2 ± 0.2	0.81
LVEDD	3	6.3 ± 0.7	6.2 ± 0.6	0.96
	7	5.7 ± 0.4	5.6 ± 0.5	0.99
	10	5.4 ± 0.4	6.2 ± 0.3	0.06
LVESD	3	2.7 ± 0.3	3.4 ± 0.6	0.13
	7	2.6 ± 0.2	2.9 ± 0.4	0.82
	10	2.4 ± 0.2	3.3 ± 0.9	**0.04**
LVEF	3	89.6 ± 1.1	80.4 ± 5.1	**0.03**
	7	87.2 ± 2.1	82.6 ± 4	0.45
	10	89.8 ± 2.1	81.4 ± 10.8	**0.05**
FS	3	56.1 ± 1.9	41.3 ± 6.5	**0.003**
	7	50.8 ± 5.7	46.9 ± 5.4	0.71
	10	55.6 ± 3.5	46.5 ± 10.6	0.09

*p*-values wrtten in bold were significant

### DOX treatment induces oxidative stress and activates autophagy in rat cardiac muscle

**Fig. 3:**
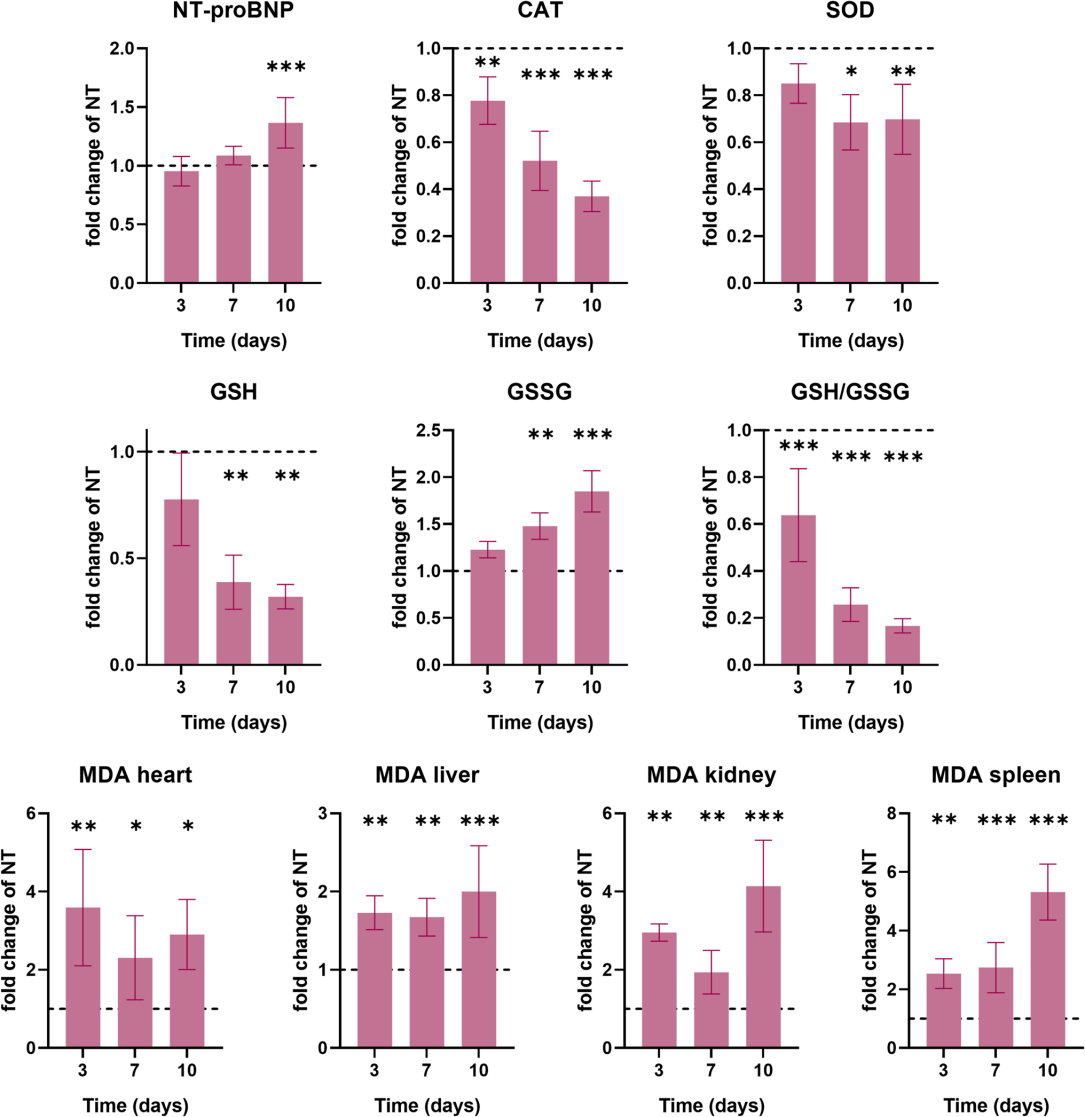
Oxidative stress after a single dose of DOX. The DOX-treated group is presented as fold change in respect to their untreated counterpart which is represented by the dotted line. NT, untreated group; DOX, doxorubicin; NT-proBNP, N-terminal pro-brain natriuretic peptide; CAT, catalase; SOD, superoxide dismutase; GSH, glutathione; GSSG, glutathione disulfide; MDA, malondialdehyde. *p < 0.05; **p < 0.01; ***p < 0.001

DOX treatment induced a depletion of GSH with a consequent increase in GSSG, reaching statistical significance after 7 days suggesting the development of a profound oxidative imbalance after a single dose of DOX, which intensified over time (Fig. 3). Concomitantly, catalase and SOD, which are major enzymes protecting from free-radicals attack, showed a significant decrease in time also after 3 and 7 days of DOX administration, respectively. The analysis of MDA, a marker of lipid peroxidation, revealed an increase in the heart homogenates of DOX-treated rats. The analysis of other organs (liver, spleen, kidneys) also showed a marked increase of MDA levels even after only 3 days, suggesting that DOX-induced organ damage was an early event which continued to be maintained in time even after a single dose (Fig. 3).

**Fig. 4:**
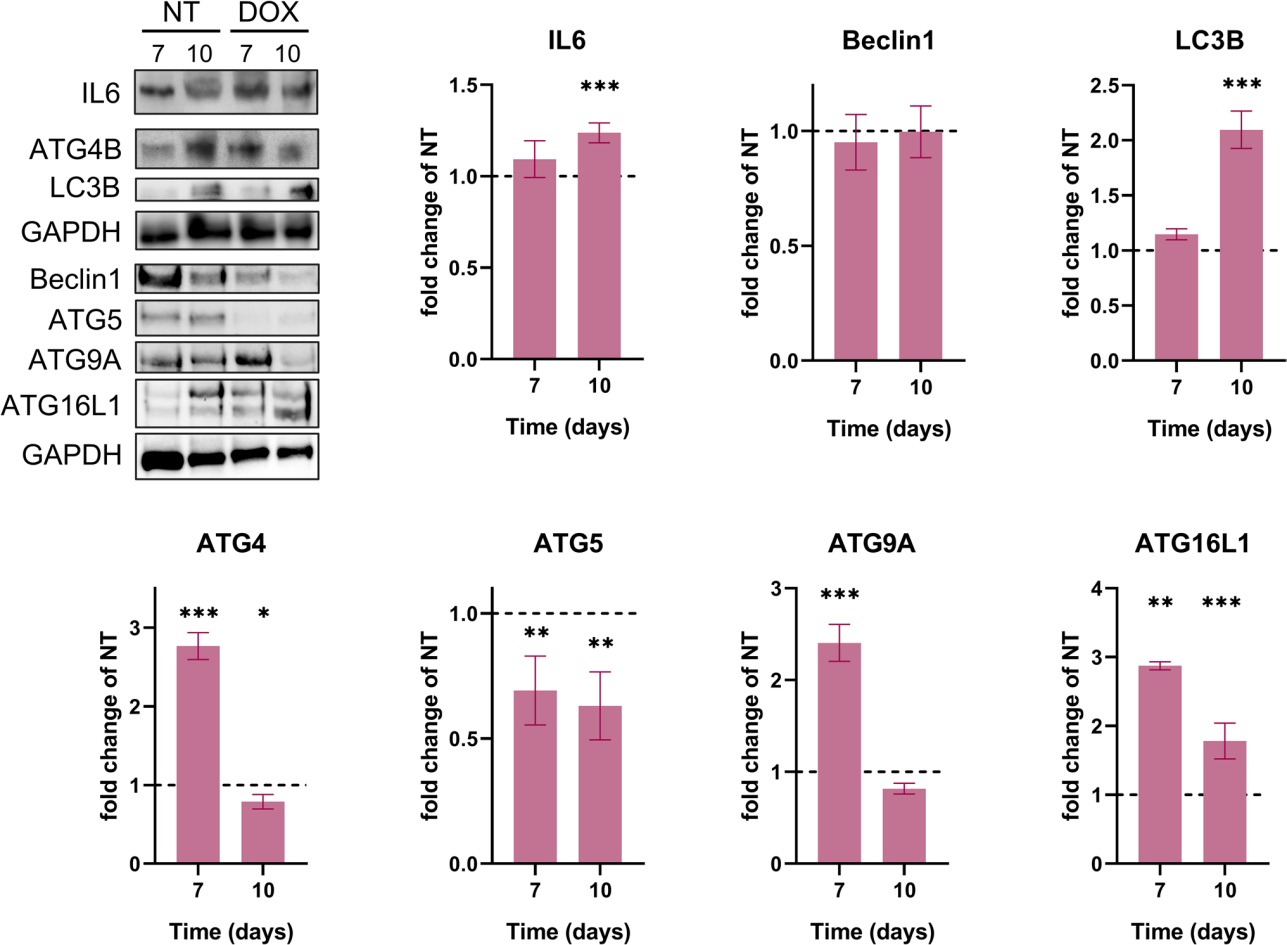
Autophagy and inflammation markers 7 and 10 days after DOX treatment. Treated samples are represented as fold change with respect to their untreated control which is represented by the dotted line. GAPDH was used as a loading control. All proteins were normalized to GAPDH. NT, untreated group; DOX, doxorubicin; *p < 0.05; **p < 0.01; ***p < 0.001

Concomitantly with these changes, DOX treatment led to a strong activation of the autophagic pathway in the treated rats’ hearts. Substantial increase in ATG16L1 (*p*<0.001) and LC3B (*p*<0.001) proteins after DOX administration together with a significant reduction in ATG5 (*p*<0.01) protein levels suggest a profound alteration and dysregulation of the autophagic response. The behavior of ATG4 and ATG9A on the other hand was oscillating, showing a strong increase at 7 days (*p*<0.001) followed by a drop at 10 days. Moreover, we observed a significant increase of IL-6 levels 10 days after DOX administration (Fig. 4).

## Discussion

Our study showed that oxidative stress and autophagy as well as inflammation are found in the presence of structural changes of AIC. Doxorubicin cardiotoxicity is of major concern to oncologists and is considered the main restriction for the clinical application of DOX (Hengel et al. 2006). In clinical practice, ECG is commonly used to diagnose heart injuries and also in the case of patients undergoing DOX therapy (Kinoshita et al. 2021). The acute cardiotoxicity of DOX is characterized by arrhythmias and conduction abnormalities (Bhagat and Kleinerman 2020; Cai et al. 2019). According to previous studies, the duration of the QRS complexes and S-T segment changes are the most reliable ECG parameters in the assessment of DOX-induced cardiotoxicity in rats (Wu et al. 2018). In this work, we showed that 7 days after DOX exposure, there was an increased proarrhythmic risk translated by an increase in the QTc interval compared to the control group. DOX has been consistently shown to widen the QT interval to a significant extent (Benjanuwattra et al. 2020). Moreover, this effect of DOX on the QT interval has been reported as the earliest abnormality detectable by ECG assessment. On the other hand, prolonged QT interval was shown by previous research to be significantly associated with left ventricular dysfunction (Benjanuwattra et al. 2020). In fact, we showed that relevant signs of cardiotoxicity on US were present already 3 days after a single dose of DOX administration in a rat model. The LV function indices, including LVEDd, LVESd, EF, and FS, were measured by transthoracic echocardiography to estimate global myocardial contractile function. We showed that at 3 days following DOX administration, the global cardiac function was significantly altered, and early signs of cardiac remodeling could also be seen in terms of thinning of the LV walls both in systole and diastole. However, the effect on cardiac function was transitory, and at 7 days, the myocardial contractility regained some of its normal function, rendering the differences between groups insignificant. On the other hand, at the 10 days time point, we found a consistent drop in the EF and a consistent increase in the LV diameters, the results confirming that DOX treatment significantly caused left ventricle dilation and seriously impaired the diastolic and systolic myocardial performance. Our data supported previous findings in which DOX administration significantly resulted in LV dysfunction confirmed by decreased SF and EF in the US assessment (Hydock et al. 2009). However, other authors have observed unchanged systolic function 2 days after doxorubicin treatment. These different results are probably related to differences in doxorubicin dose (25 mg vs 20 mg), evaluation periods, and animal models (mice vs rats) (Kizaki et al. 2006). Although there was a general trend toward abnormal ECG and US findings indicative of cardiac damage in DOX-treated rats, they were not significant in all studied time points suggesting that the changes are dynamic and might be reversible at least to some extent. It was previously demonstrated in rats that 12 weeks after a single treatment dose was a critical time point in the course of the development of anthracycline-induced cardiotoxicity in the rat (Yeung et al. 2002). This is the time point at which the modified cardiac output in treated animals has stabilized, and the decline in cardiac output has been clearly established. However, since reports studying changes during the first days are scarce, a full evaluation of cardiac plasticity treatment over time is yet to be fully understood.

To delve into the mechanisms involved in the cardiac alterations induced by DOX treatment, we evaluated the level of oxidative stress in the heart muscles. An altered membrane function due to DOX-induced lipid peroxidation might be responsible for most of the ECG changes as it is well-known that DOX induces cardiomyocyte damage through various mechanisms including ROS generation (Nordgren and Wallace 2020). GSH is one of the most important cellular ROS scavengers, and it is oxidized to GSSG in oxidative stress conditions leading to the alteration of the GSH:GSSG ratio (Zitka et al. 2012). Catalase overexpression has been shown to protect cardiomyocyte contractility (Ye et al. 2004), so the fact that DOX treatment can induce an acute depletion with the consequent increase in oxidative stress might contribute to the development of muscle weakness and the decrease of ventricular ejection and signs of cardiac dilatation. The same pattern of evolution was seen in the SOD activity, in the heart tissue suggesting a profound redox imbalance induced by DOX. Lipid peroxidation is the result of oxidative damage action on lipids containing carbon–carbon double bonds, with the generation of a wide variety of aldehydes, such as MDA. In normal circumstances, low levels of lipid peroxidation are curbed by the cellular antioxidant defense systems. In contrast, when the level of damage overwhelms the adaptative capacity of the cells, apoptosis and necrosis cascades are triggered (Ayala et al. 2014). In this study, we showed that not only was DOX able to cause a significant imbalance of the redox status, but the extent of this damage was sufficient to induce lipid peroxidation, as revealed by the high systemic MDA levels found in the heart, liver, kidneys, and spleen. Moreover, this damage was also associated with a significant inflammatory response as shown by the increase of IL-6, 10 days after DOX exposure. Previous studies have also shown that IL-6 increases in response to DOX, but its role in myocardial oxidative stress induced by AIC and the regulatory mechanism still remains to be clarified (Jiang and Zhang 2020).

Autophagy, a multistep biological process highly conserved in eukaryotic cells, is an essential physiological process for the maintenance of cellular homeostasis (Chun and Kim 2018). The complex balance between the promotion and inhibition of autophagy can differentiate between cellular survival and death (Xiao et al. 2019). Its dysregulation under severe stress conditions can lead to the development of cardiac dysfunction and cause heart failure (“Doxorubicin-induced cardiotoxicity,” n.d.). In this study, we show that autophagy is altered, just 1 week after a single DOX injection in rats. LC3B and ATG16L1 proteins showed a significant upregulation in DOX-treated hearts as previously reported by other authors (Katamura 2014; Li et al. 2016). LC3B expression levels have been shown to be increased by DOX through the inhibition of Bcl-2 (Kobayashi et al. 2010) suggesting that cardiomyocyte death is due to the DOX-induced activation of apoptosis. The regulatory pathways of autophagy remain to be fully elucidated, also regarding the role of DOX in the regulation of autophagy in cardiac cells.

Furthermore, to confirm the extent of these DOX-induced alterations, we measured serum NT-proBNP which is a widely used marker in clinical medicine as it not only reflects the presence of cardiac dysfunction but also indicates its severity (Kittiwarawut et al. 2013). DOX-treated rats showed a significant increase in NT-proBNP levels 10 days after treatment. This was a week after the first signs of oxidative stress and autophagy dysregulation began, thus suggesting the impossibility of cardiomyocytes to mitigate the damaging effects of DOX and the initiation of clinically significant cardiac damage and remodeling as yielded also by the ECG and US findings. Even though the measurement of NT-proBNP is still debated in the case of patients with DOX-induced cardiotoxicity, it remains an important, highly used marker for heart failure even in clinical trials as its increase is related to early cardiac injury (Advani et al. 2017; Mladosievicova et al. 2012; Rüger et al. 2020).

DOX-induced molecular alterations in the cardiac muscle could still occur in clinically asymptomatic patients, as the functional impact can be too subtle to detect (Sulaiman et al. 2021), leading to a progressive weakening of the heart muscle which may appear much later in life. Strategies for the prevention of DOX-induced cardiotoxicity are already being used in oncological patients. For example, the concomitant administration of dexrazoxane, a ROS-reducing agent, successfully protected against cardiac decompensation (Ganatra et al. 2019; Macedo et al. 2019). However, clinical practice guidelines support its use only in specific patients (Leong and Lenihan 2022), possibly excluding many others who might also benefit from it. As far as targeting autophagy is concerned, an optimal strategy has yet to be discovered, as the administration of anti-autophagic drugs, such as chloroquine, were also found to induce ECG changes and even cardiomyopathy (Kimura et al. 2013; Yogasundaram et al. 2018). Thus, the identification of well-defined, specific targets as well as the optimal drugs is still under investigation, and further studies on this topic are still required.

In conclusion, the current study showed that the perturbances in redox balance, inflammatory, and apoptotic processes caused by a single dose of DOX were major. ECG and US investigations as early as 3 days after DOX exposure revealed cardiac dysfunction validated by a significant drop in LVEF, an increased proarrhythmic risk as showed by the increased QTc interval, as well as signs of cardiac remodeling. Even though these changes were dynamic, they yield a possible treatment window for the prevention of cardiac damage and may offer the possibility of avoiding DOX treatment toxicities.

## Data Availability

All data generated in this study was reported in the paper.
